# Rapid-onset Severe Cytokine Release Syndrome With Marked Interleukin-6 Increase and Acute Liver Injury After the First Tarlatamab Dose in SCLC: Case Report

**DOI:** 10.1016/j.jtocrr.2025.100917

**Published:** 2025-10-10

**Authors:** Kento Takagi, Go Saito, Toshiaki Inazaki, Hikaru Shojima, Jun Miyakoshi, Akira Naito, Shun Sato, Takashi Shimazui, Haruka Anzai, Chiaki Imai, Takuji Suzuki

**Affiliations:** a Department of Respirology, Graduate School of Medicine, Chiba University, Chiba, Japan; b Department of Emergency and Critical Care Medicine, Graduate School of Medicine, Chiba University, Chiba, Japan; c Department of Gastroenterology, Graduate School of Medicine, Chiba University, Chiba, Japan; d Division of Pharmacy, Chiba University Hospital, Chiba University, Chiba, Japan

**Keywords:** Tarlatamab, SCLC, Cytokine release syndrome, Liver injury, Case report

## Abstract

Tarlatamab is a novel bispecific T-cell engager therapy with promising efficacy in patients with previously treated extensive-stage SCLC. Cytokine release syndrome (CRS) is the most common adverse event related to tarlatamab, although severe CRS remains rare, and grade 3 or higher adverse events have been reported to be less common with tarlatamab than with chemotherapy. Available clinical data on severe adverse events associated with tarlatamab remain limited. Herein, we report a case of a 55-year-old woman with extensive-stage-SCLC who was treated with tarlatamab. Severe CRS and liver injury rapidly developed in the patient after the first tarlatamab dose, which led to treatment discontinuation. This report also presents the temporal changes in serum interleukin-6 levels, highlighting its potential utility as a biomarker for the onset and severity of CRS.

## Introduction

SCLC is an aggressive malignancy with a poor prognosis, and the treatment options are particularly limited in the second-line setting and beyond. Tarlatamab, a novel bispecific T-cell engager therapy targeting delta-like ligand 3, has shown promising efficacy in patients with previously treated extensive-stage (ES)-SCLC.^1^^,^^2^ Cytokine release syndrome (CRS) is the most common tarlatamab-related adverse event, occurring in approximately 55% of patients.^1^, ^2^, ^3^ Nevertheless, grade ≥3 CRS is uncommon, with reported incidence rates of approximately 1%.^1^, ^2^, ^3^ Moreover, the reported overall incidence of grade 3 or higher adverse events is lower with tarlatamab than with chemotherapy.^2^ Thus, clinical data on severe adverse events associated with tarlatamab remain limited. Herein, we present a case of ES-SCLC with severe CRS and liver injury after the first tarlatamab dose.

## Case Presentation

The patient was a 55-year-old woman diagnosed with ES-SCLC who presented with multiple liver and bone metastases. She received first-line combination therapy with carboplatin, etoposide, and durvalumab, the best response of stable disease being achieved. After four cycles (approximately 3 months), liver metastases progressed, and amrubicin was initiated as second-line therapy. After six cycles (approximately 5 months), despite stable disease as the best response, liver metastases further worsened; new brain metastases emerged, and tarlatamab was administered as third-line therapy. Four hours after the initial 1-mg tarlatamab dose, fever developed in the patient. Subsequently, 400 mg of acetaminophen was administered orally. Ten hours after tarlatamab administration, hypotension developed in the patient at 84/52 mmHg. At 11 hours after administration, blood tests revealed a grade 4 increase in aspartate aminotransferase (AST) from 32 to 689 U/L (upper limit of normal [ULN], 30 U/L) and a grade 3 increase in alanine aminotransferase (ALT) from 23 to 456 U/L (ULN, 23 U/L) according to the Common Terminology Criteria for Adverse Events version 5.0. In addition, ferritin increased from 102.4 to 830.1 ng/mL, and interleukin-6 (IL-6) was markedly increased to 29,321 pg/mL (baseline unavailable). On the basis of the diagnosis of CRS, 8 mg of dexamethasone was administered intravenously with fluid therapy. Nevertheless, the patient’s blood pressure remained low, at 72/59 mmHg. After the diagnosis of grade 3 CRS according to the American Society for Transplantation and Cellular Therapy Consensus Grading, continuous norepinephrine infusion was initiated, and tocilizumab (320 mg, 8 mg/kg) was administered. Despite two additional doses of dexamethasone, vasopressor-dependent hypotension persisted. The patient was admitted to the intensive care unit on day 2 after tarlatamab administration.

**Figure 1:**
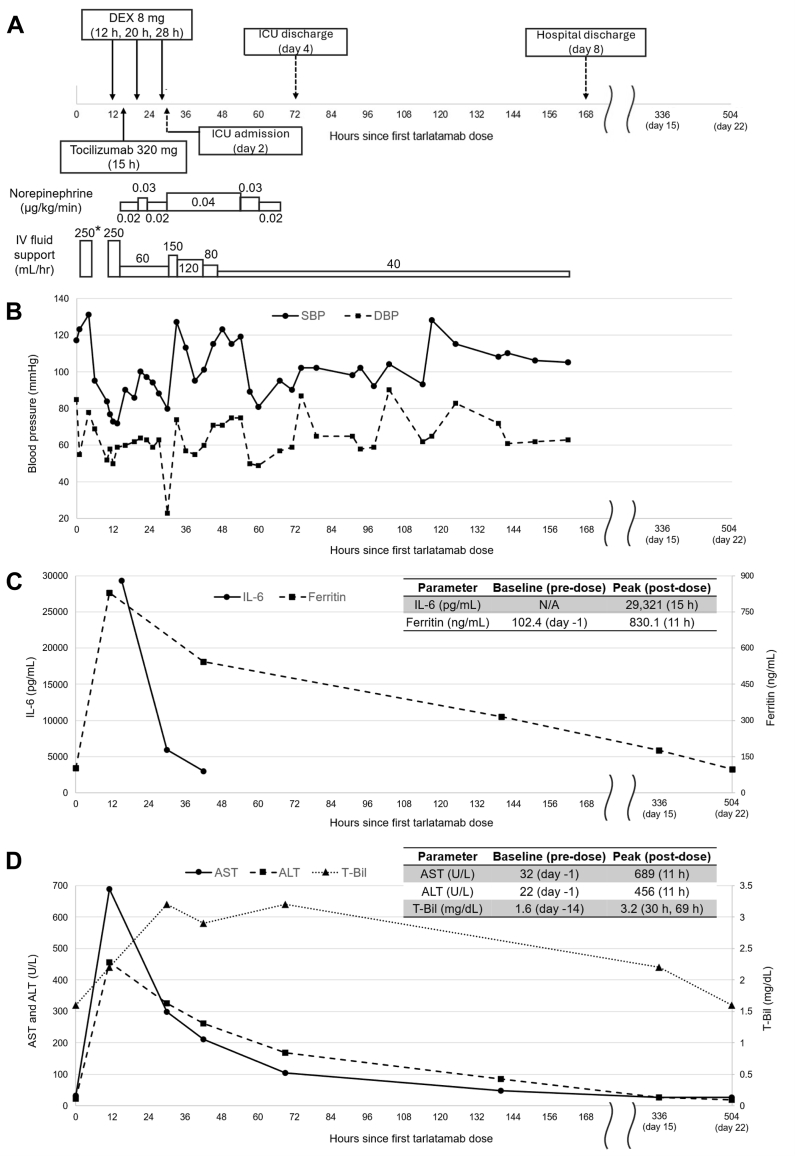
Clinical course showing the temporal changes in blood pressure, inflammatory markers, and liver function parameters in the present case. (*A*) Key therapeutic interventions and clinical events. (*B–D*) Time course of blood pressure, inflammatory markers (interleukin-6 and ferritin), and liver function parameters (AST, ALT, and T-Bil). (*C* and *D*) Baseline and peak values of each parameter after tarlatamab administration. Note: In this figure, time 0 represents the time of tarlatamab administration. For clarity, the x-axis uses different time scales for the period before and after hospital discharge. The wavy line on the x-axis denotes a discontinuous timescale. ∗ The initial 250 mL represents a routine infusion of 1000 mL saline given more than 4 hours after tarlatamab administration. Other fluid support volumes represent therapeutic interventions. ALT, alanine aminotransferase; AST, aspartate aminotransferase; DBP, diastolic blood pressure; DEX, dexamethasone; ICU, intensive care unit; IL-6, interleukin-6; IV, intravenous; N/A, not available; SBP, systolic blood pressure; T-Bil, total bilirubin.

Figure 1*A*–*D* shows the clinical course, including the temporal changes in blood pressure, inflammatory markers (IL-6 and ferritin), and liver function parameters (AST, ALT, and total bilirubin). Inflammatory marker levels gradually improved, and blood pressure progressively recovered with circulatory support using fluids and norepinephrine. By day 4 after tarlatamab administration, circulatory support was discontinued, and the patient was discharged from the intensive care unit. Regarding liver injury, Common Terminology Criteria for Adverse Events grade 2 hyperbilirubinemia was observed (total bilirubin increased from 1.6 to 3.2 mg/dL [ULN, 1.5 mg/dL]), along with increased AST and ALT levels. No alternative causes of liver injury were identified after evaluation of thyroid function, autoantibodies, and viral serologies. All abnormalities gradually resolved with treatment for CRS and supportive care.

**Figure 2:**
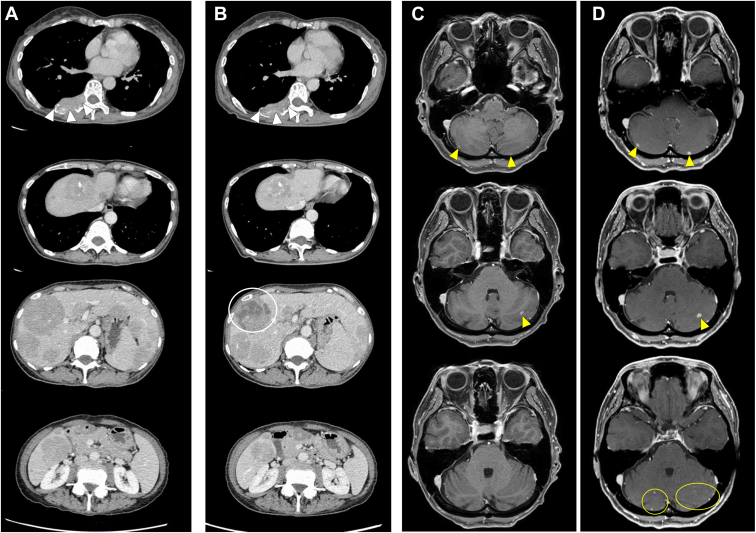
Contrast-enhanced computed tomography and brain magnetic resonance imaging before and after tarlatamab administration. Contrast-enhanced computed tomography images (*A*) before and (*B*) on day 15 after tarlatamab administration. The right inferior rib metastasis (white arrowheads) and multiple liver metastases show reduction in size. In addition, non-enhancing areas suggestive of necrosis are observed within some of the liver lesions (white circle). Brain magnetic resonance imaging (*C*) before and (*D*) on day 50 after tarlatamab administration. Enlargement of existing cerebellar metastases (yellow arrowheads) and the emergence of new multiple cerebellar metastases (yellow circle) are observed.

**Figure 3:**
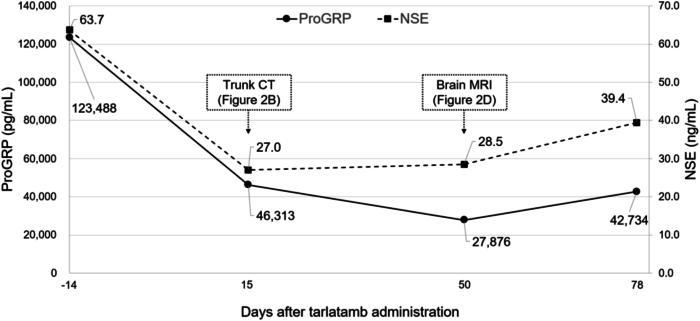
This graph illustrates the trends of tumor marker levels, pro–gastrin-releasing peptide (ProGRP) and neuron-specific enolase (NSE), after tarlatamab administration. Baseline levels were measured 14 days before tarlatamab administration. Both ProGRP and NSE decreased after tarlatamab administration, consistent with the radiologic improvement observed on trunk computed tomography. Nevertheless, these levels subsequently increased in association with progression of brain metastases. CT, computed tomography; MRI, magnetic resonance imaging.

Considering the severe CRS and liver injury after the first dose of only 1 mg, tarlatamab was discontinued from day 8. Computed tomography on day 15 after tarlatamab administration revealed a decrease in lesion size, including multiple liver metastases (Fig. 2*A* and *B*), with tumor marker levels decreasing in parallel. Nevertheless, brain magnetic resonance imaging on day 50 revealed progression of brain metastases (Fig. 2*C* and *D*). During subsequent follow-up, these levels began to increase again (Fig. 3). Nevertheless, the patient’s general condition remained stable up to day 106, the latest follow-up available.

## Discussion

To the best of our knowledge, this is the first report of severe CRS with concomitant liver injury occurring shortly after an initial tarlatamab dose for ES-SCLC. The markedly increased serum IL-6 levels at CRS onset provide clinically meaningful insights.

In the present case, the development of CRS was accompanied by markedly increased IL-6 levels. Data from earlier phase trials of tarlatamab have suggested a positive correlation between the incidence of CRS and peak serum IL-6 levels.^4^ Furthermore, in chimeric antigen receptor T-cell therapy, which, like bispecific T-cell engagers, engages cluster of differentiation 3 to activate T cells, a median peak IL-6 level of 8309 pg/mL has been reported in patients with grade 4 to 5 CRS, compared with 122 pg/mL in those with grade 1 to 3 CRS or no CRS.^5^ These findings further support an association between peak IL-6 levels and severity of CRS. Notably, the peak IL-6 levels in the present case exceeded those reported for grade 4 to 5 CRS after chimeric antigen receptor T-cell therapy. Clarifying the role of IL-6 in tarlatamab-induced CRS may be essential for identifying early biomarkers and guiding management strategies.

A high liver tumor burden may have been associated with the acute liver injury observed in this case. Given that delta-like ligand 3 is minimally expressed on the surface of normal cells, including hepatocytes, tarlatamab-induced liver dysfunction is more likely to be secondary to CRS rather than due to direct cytotoxicity. In particular, multiple liver metastases in this case may have contributed to the extensive local cytokine release within the liver, potentially leading to rapid hepatic injury. Notably, despite the presence of liver metastases in more than 30% of participants in a phase 2 trial of tarlatamab, liver enzyme increases were reported in approximately 10% of cases, mostly grade 1 to 2 with no grade 4 events.^1^^,^^3^ Similarly, liver enzyme increases did not seem to occur in more than 10% of patients in a phase 3 trial.^2^ These findings suggest that not only the presence but also the number and size of liver metastases may be related to the risk of tarlatamab-induced hepatotoxicity. Further studies are warranted to identify additional risk factors, including those associated with patients without liver metastases.

## Conclusion

We encountered a case of ES-SCLC that rapidly developed into CRS and liver injury after the first tarlatamab dose. Clinical data on grade 3 or higher adverse events associated with tarlatamab, including CRS, remain limited. Further research is needed to clarify the risk factors for severe toxicities.

## CRediT Authorship Contribution Statement

**Kento Takagi**: Data curation, Investigation, Writing - original draft.

**Go Saito**: Conceptualization, Investigation, Writing - review & editing.

**Toshiaki Inazaki:** Investigation, Validation.

**Hikaru Shojima:** Investigation, Validation.

**Jun Miyakoshi:** Investigation, Validation.

**Akira Naito:** Investigation, Validation.

**Shun Sato:** Investigation, Validation.

**Takashi Shimazui:** Investigation, Validation.

**Haruka Anzai:** Investigation, Validation.

**Chiaki Imai:** Investigation, Validation.

**Takuji Suzuki**: Supervision.

## Declaration of Generative AI and AI-Assisted Technologies in the Writing Process

During the preparation of this work, the authors used ChatGPT (OpenAI) to edit and refine English expressions for clarity and fluency. After using this tool, the authors reviewed and edited the content as needed and take full responsibility for the content of the publication.

## Disclosure

Dr. Takagi reported personal fees from AstraZeneca, Chugai Pharmaceutical, Taiho Pharmaceutical, and Eisai outside the submitted work. Dr. Saito reported personal fees from Ono Pharmaceutical, Chugai Pharmaceutical, AstraZeneca, Novartis, MSD, Pfizer, Daiichi Sankyo, and Taiho Pharmaceutical outside the submitted work. Dr. Suzuki reported personal fees from AstraZeneca and Boehringer Ingelheim outside the submitted work. The remaining authors declare no conflicts of interest.
